# Green Deep Eutectic Solvents for Microwave-Assisted Biomass Delignification and Valorisation

**DOI:** 10.3390/molecules26040798

**Published:** 2021-02-04

**Authors:** Giorgio Grillo, Emanuela Calcio Gaudino, Roberto Rosa, Cristina Leonelli, Ana Timonina, Saulius Grygiškis, Silvia Tabasso, Giancarlo Cravotto

**Affiliations:** 1 Dipartimento di Scienza e Tecnologia del Farmaco, University of Turin, Via Giuria 9, 10125 Turin, Italy; giorgio.grillo@unito.it (G.G.); emanuela.calcio@unito.it (E.C.G.); 2 Dipartimento di Scienze e Metodi dell’Ingegneria, Università degli Studi di Modena e Reggio Emilia, Via Amendola 2, 42122 Reggio Emilia, Italy; roberto.rosa@unimore.it; 3 Dipartimento di Ingegneria “Enzo Ferrari”, Università degli Studi di Modena e Reggio Emilia, Via Vivarelli 10, 41125 Modena, Italy; cristina.leonelli@unimore.it; 4 JSC Biocentras, LT-14117 Vilnius, Lithuania; ana.timonina@supernode.lt (A.T.); grigiskis@biocentras.lt (S.G.); 5 Dipartimento di Chimica, University of Turin, Via P. Giuria 7, 10125 Turin, Italy

**Keywords:** NaDES, microwave-assisted delignification, dielectric properties, lignin-derived phenolics, enzymatic hydrolysis

## Abstract

Aiming to fulfil the sustainability criteria of future biorefineries, a novel biomass pretreatment combining natural deep eutectic solvents (NaDESs) and microwave (MW) technology was developed. Results showed that NaDESs have a high potential as green solvents for lignin fractionation/recovery and sugar release in the following enzymatic hydrolysis. A new class of lignin derived NaDESs (LigDESs) was also investigated, showing promising effects in wheat straw delignification. MW irradiation enabled a fast pretreatment under mild condition (120 °C, 30 min). To better understand the interaction of MW with these green solvents, the dielectric properties of NaDESs were investigated. Furthermore, a NaDES using the lignin recovered from biomass pretreatment as hydrogen bond donor was prepared, thus paving the way for a “closed-loop” biorefinery process.

## 1. Introduction

In the last decade a growing awareness of solvents impact on environmental pollution, prompted a vast search of greener and more sustainable alternatives [1]. Simultaneously, owing to the increasing environmental concerns, biomass conversion to fuels and valuable platform chemicals has attracted remarkable attention over traditional petroleum-based industrial process, according to a biorefinery approach [2,3].

**Figure 1:**
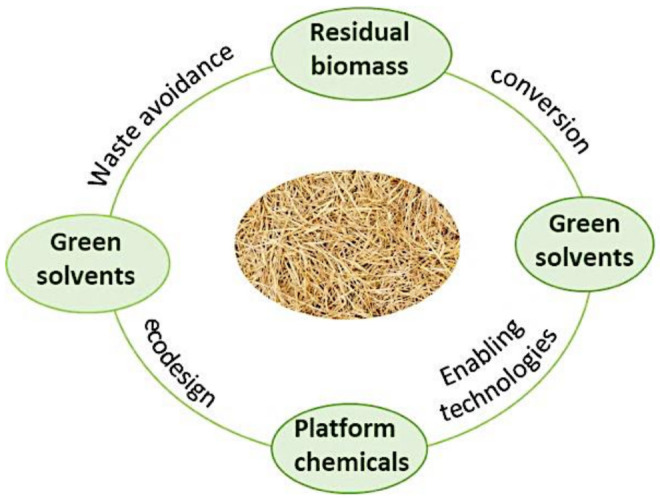
Circular economy in the biomass valorisation using biomass-derived green solvents.

Lignocellulosic biomass is an easily available, inexpensive and renewable resource, mainly composed of three biopolymers, namely cellulose, hemicellulose and lignin. Currently, the most promising commercially viable route for lignocellulosic biomass valorisation is fermentation of cellulose and hemicellulose sugars into ethanol [4]. A conventional process design for biomass valorisation includes biomass size reduction, pretreatment, enzymatic hydrolysis, fermentation and distillation as major process steps. Moreover, the valorisation of the co-obtained lignin is advisable for a profitable biomass conversion, [5]. Indeed, being the conversion of lignocellulose to ethanol a complex and expensive process, the cost of the ethanol obtained cannot compete in the market. However, a promising strategy to lower the production cost of cellulosic ethanol is a biorefinery development, which entails the valorisation of all the components of biomass through the production of chemicals besides ethanol, according to a zero-waste approach [4]. However, owing to biomass recalcitrance, due to cellulose-lignin interactions, harsh pretreatment conditions (strong acids, high temperature, long process time, etc.) are required to obtain cellulose-rich residue. Moreover, the high costs associated may hamper the application [6,7]. It has been estimated that the biomass pretreatment step still remains the most expensive biomass process, around 20% of the cost of biofuel production [8]. One way to overcome this limitation could be the combined use of sustainable and renewable solvents with enabling technologies in order to reduce the overall process time and energy consumption. While conventional thermal processes remain popular at both research and industrial levels, microwaves (MWs) draw increasing attention in view of its potential for selective activation of substrate and lower energy demand [3,9]. Moreover, recent developments in synthetic chemistry, biotechnology and chemical engineering are leading to a new concept of biomass conversion based on the use of biomass-derived solvents, in order to describe a “closed-loop” biorefinery process (as illustrated in Figure 1) in the frame of a circular economy perspective [10].

Solvents derived from biomass (especially hemicellulose and lignin) including γ-valerolactone (GVL) [11,12], 2-methyltetrahydrofuran [13], tetrahydrofuran (THF) [14], ionic liquids (ILs) [15] and deep eutectic solvents (DESs) [16,17] have been successfully applied in biomass pretreatment and conversion.

In particular, ILs have gained greater attention as alternative solvents for biomass pretreatment, because the ability of some of them to dissolve cellulose or make it amorphous [18]. Moreover, their non-volatility (their melting points are generally below 100 °C) [19] generated safer applications, in particular with tailor-made solvents for organic synthesis [20,21] and biomass extraction and conversion [22]. For example, high temperatures can be employed without the use of expensive pressurized systems, meanwhile avoiding the emissions of solvents vapours. Nevertheless, ILs synthesis showed high production costs of their individual components. With physical and chemical properties comparable to ILs, deep eutectic solvents (DESs) recently emerged as a potential greener alternative for several chemical and biological applications [23]. Typically, DESs are prepared by combining hydrogen bonding donors (HBDs) and hydrogen bonding acceptors (HBAs) to form a eutectic mixture with a final melting point much lower than the individual components [24]. DESs are favoured over conventional ILs in terms of the availability and costs of raw materials and the ease of storage and synthesis [25].

According to some reported estimations, the cost to synthesize DES was only 20% of that of ILs and the DES components were ten times less expensive than those of ILs [26,27]. The application of DESs as an alternative to ILs in dissolving the polysaccharides and their constituents (i.e., cellulose, xylose, arabinose, starch and chitin) and lignin present in biomass has attracted a huge interest to produce biofuels, value added products and commodity chemicals [28,29]. Recycling and reuse of DESs are one of the major advantages for its application in low-cost high-volume industrial applications like biomass processing [30]. DESs can be recycled more readily than ILs because their synthesis/regeneration does not involve any chemical reactions since it only entails the formation or breaking of the hydrogen bonding network that binds the HBD and HBA components. The recycling of DESs in pretreatments’ studies has been rarely investigated but broadly acknowledged as a pressing research need for the commercial viability of the biomass based biorefinery. Recently, Kim et al. [16] have evaluated the recovery, reuse and efficacy of the DESs during the pretreatment of switchgrass.

Recently, a new type of deep eutectic solvents, known as natural deep eutectic solvents (NADESs) have gained great interest as greener and more efficient alternatives to conventional organic solvents for biomass pretreatment steps [31]. NADESs are commonly composed of two or more naturally occurring, non-toxic and inexpensive compounds, which are bounded together through hydrogen bonding [32]. These were mainly composed of primary metabolites, such as amino acids and sugars, which are abundantly present as ubiquitous natural compounds. Nearly hundreds of different hydrophilic and hydrophobic NADESs were prepared (mainly based on choline chloride, ChCl, as HBA) foreseeing their potential uses for several unknown and still untapped areas of applications. From an environmental and economic perspective, NADESs offer many striking advantages including biodegradability, sustainability, low costs and simple preparation. In particular, due to their non-toxicity and the ability to dissolve both non-polar and polar compounds, NADESs have been used as efficient and safe solvents to dissolve DNA [32,33] as media for enzymatic reactions [34], biotransformations [35], extraction of phenolics [36,37,38] and for biomass processing and stabilization of natural pigments [39]. However, the physicochemical properties of most NADESs and the relationship between their molecular composition and the solvent properties of the resulting eutectic mixtures are not fully understood. Furthermore, most NADESs share some limitations already observed in conventional synthetic ILs and DESs: their high viscosity (typically 200–500 cP at 40 °C) is the most obvious issue, which leads to some practical problems, including time-consuming solvent transfer operations and slow mass transfer in dissolutions or extractions. In the case of ILs, this is generally partly overcome by applying external physical forces, such as MW irradiation and stirring at high temperature, thus accelerating the dissolving process. Addressing these critical issues by coupling MW technologies with NADESs for biomass pretreatment could make the biomass delignification processes more feasible, thus avoiding the strong negative effects of lignin on cellulase action during enzymatic breakdown of complex cellulose molecules into simple reducing sugars.

Several recent reviews highlight the potential use of NADESs for biomass pretreatment, [40,41,42]; in particular, Kumar et al. (2016) [43] recently demonstrated how hydrophilic NADESs could replace other harsh chemical agents such as hydrochloric acid and sodium hydroxide for pretreatment of lignocellulosic agriresidues.

Moreover, when combining NADESs with enabling technologies (i.e., ultrasound irradiation) [17,44] an efficient wheat straw (WS) delignification can be achieved. This effect is due to the reduction of solvent viscosity and the consequent improved mass transfer resulting by the generation of high-energy microenvironments.

Kim et al., in 2018, [16] developed a new class of renewable lignin-derived NADESs (LigDESs), combining the use of ChCl (as HBA) with some lignin-derived phenols (as HBDs) like 4-hydroxybenzyl alcohol (ChCl:HBA), catechol (ChCl:CAT), vanillin (ChCl:VAN) and *p*-coumaric acid (ChCl:PCA) to produce a new eutectic mixture suitable for delignification of switchgrass. Therefore, in this paper, some NADES, including lignin-derived ones, will be tested in combination with MW technology, to improve the delignification of wheat straw for further enzymatic conversion. In addition, a new NADES obtained using the products recovered after lignin precipitation will be exploited, according to a circular approach.

## 2. Results and Discussion

### 2.1. Natural Deep Eutectic Solvents (NaDES) and Lignin-Derived Deep Eutectic Solvents (LigDES)

A set of four different NaDESs was chosen to evaluate the impact of those solvents on WS delignification under MW irradiation. In particular, keeping ChCl as HBA, four different HBDs were screened, tailoring the system behaviour. Lactic acid (LA), glycerol (Gly) and ZnCl_2_ (Zn) were selected in order to provide different acidity to NaDES, according to the bond-donors nature.

Similarly, different LigDESs were tested, studying three different lignin-derived model compounds as HBD, namely 4-hydroxybenzyl alcohol (4Hba), catechol (Cat) and eugenol (Eug) (Table 1).

The inherent viscosity of most NaDESs requires a relatively long extraction time and can lead to some difficulties in post-extraction procedures, e.g., residual solvents in the extracts interfering with the analysis. There are two ways to solve this problem, by increasing the working temperature or by adding a certain percentage of water. It has been observed that the addition of very small amounts of water can result in a dramatic decrease in the viscosity of most NaDESs. Thus, 10% of water was added only to ChZn and ChEug as suggested by Dai et al. [45] to reach a stable eutectic mixture. Furthermore, this proved to successfully decrease the viscosity.

### 2.2. Dielectric Properties of Deep Eutectic Solvents (NaDESs and LigDESs)

The synergy between NaDES and MW heating could contribute very efficiently to the biomass delignification, owing to the interaction of MW irradiation with electrolytic solvents [46]. Therefore, to better understand this kind of interaction, we measured the dielectric properties of all of the NaDESs and LigDESs used in this work, since it is well known that MW heating is strictly related to the samples’ dielectric features [8].

The irradiation frequency was swept in the range of 0.3–3 GHz, at the proposed treatment temperature, i.e., 120 °C (see SI, Figures S1–S7). The response efficiency to irradiation can be explained by the parameter *tanδ*, described in Equation (1), where the dielectric constant *ε′* indicates the ability of a material to be polarized by an external electric field and the loss factor *ε″* quantifies the efficiency with which the electromagnetic energy is converted into heat.

(1) $$tan\delta =\;\frac{\varepsilon^{\prime \prime }}{\varepsilon^{\prime }}$$

Thus, the ratio between those two parameters, describes the capability of the material to convert MW energy into heat (dielectric heating), depending on frequencies and temperatures. For dipole polarization dictates MW heating of polar molecules, higher dielectric constant values are desired for efficient dielectric heating.

**Figure 2:**
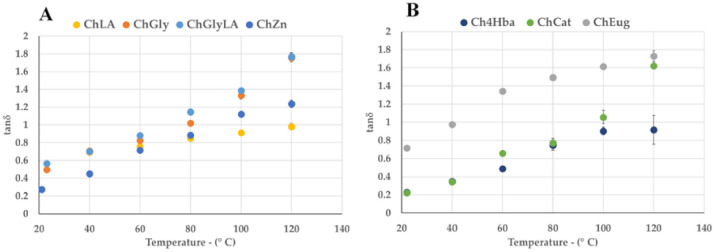
Variation of tan δ (@2.45 GHz) according to temperature. (**A**): NaDESs (ChLA, ChGly, ChGlyLA and CHZn); (**B**): LigDESs (Ch4Hba, ChCat and ChEug).

Commercially available MW-assisted reactors are usually allowed to work at 2.45 GHz. For this reason, a more detailed study was performed at this specific frequency, investigating the tan δ variation with temperature, from ambient conditions to the operating ones (120 °C). Results are reported in Figure 2A,B, for NaDESs and LigDESs, respectively, showing that all the considered NaDESs and LigDESs exhibited good dielectric properties. More in detail, tan δ values of almost all solvents increased with increasing temperatures and were higher than 1.0 at operating temperatures (120 °C), except for ChLA and Ch4Hba, which were instead slightly under this value.

These findings endorsed the hypothesis of a synergistic effect between NaDESs and MW heating, which could prompt the efficiency of the biomass pretreatment.

### 2.3. Microwave-Assisted Wheat Straw (WS) Delignification—NaDES and LigDES

The NaDESs and LigDESs previously prepared and characterized were used as solvents for MW-assisted deconstruction of wheat straw (WS) under different conditions, to obtain cellulose rich matter and lignin. Recently, Abbott et al. [47] reported that the hydroxyl groups of choline chloride forms hydrogen bonds with the cellulose and stabilizes the cellulose system; thus, dissolution of cellulose or hemicellulose is highly unlikely, proving the specificity of NADES reagents toward lignin solubilization.

**Figure 3:**
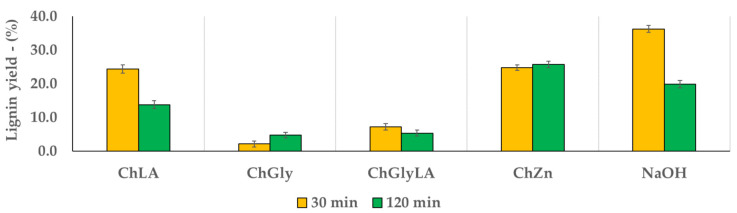
Lignin precipitations yield for different NaDES, according to process time. NaOH 10% is reported as a benchmark.

**Figure 4:**
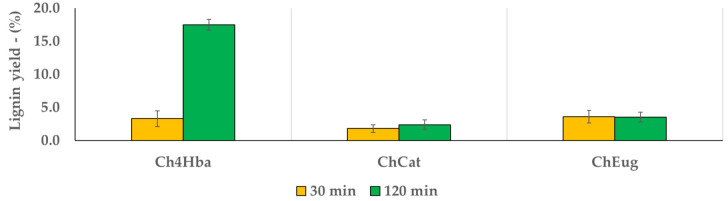
Lignin precipitation yield for different LigDES, according to process time.

Despite the biomass delignification is a time-consuming process [17], MW can dramatically reduce the operation time. For this reason, different extraction times were compared (30 and 120 min) [18]. When using MW, extraction times longer than two hours are not sustainable. The achieved results are reported in Figure 3 and Figure 4, for NaDESs and LigDESs, respectively, considering the amount of lignin recovered by precipitation after water addition to investigate the extraction yields as referred to the starting WS (dry wt%). Moreover, the same tests were performed under alkaline conditions (NaOH 10%, expressed on WS dry matter), for the sake of comparison.

Being ChCl the only HBA, the pretreatment efficiency of DES varied depending on the compounds used as HBDs (Table 1).

In particular, among the four NaDESs, ChLA and ChZn were the most efficient in terms of lignin recovery at 30 min treatment, although the yields were lower than using NaOH. However, longer treatment times afforded worse results both under alkaline conditions, but also with acidic NaDES. On the contrary, when using neutral mixtures, such as ChGly and ChZn, lignin recovery increased as the residence time increased, being the efficiency of ChZn even higher than NaOH after 120 min (Figure 3).

On the other hand, LigDESs were in general less effective in lignin extraction, as it is worthy of note only the treatment with Ch4Hba for 120 min (Figure 4). It can be hypothesised that polyols-based DESs with more hydroxyl groups (ChGly and ChGlyLA) and phenol-based DES might have formed stronger intermolecular H-bonds in the solvent system, and thus they lacked sufficient amounts of free and active groups to interact with the biomass components, resulting in lower extraction efficiency [48]. However, in-depth understanding of chemistries as hydrogen bond interactions and melting point depression of a eutectic mixture beyond DES formation is necessary to better understand the different behaviour of phenol-based DES. Therefore, the structures and properties of the HBAs and HBDs appeared to be closely related to the lignin removal capability and ultimately influenced the subsequent enzymatic polysaccharide digestion. These relationships need to be further addressed.

### 2.4. Antioxidant Activity of Recovered Liquid Fraction—Pre- and Post-lignin Precipitation

After MW-assisted treatment, the obtained extracts were tested for their antioxidant activity by means of the 2,2-diphenyl-1-picrylhydrazyl (DPPH) assay. Results, expressed as the half maximal effective concentration or amount of compound/extract necessary to decrease the initial concentration of DPPH to 50% at equilibrium (EC50), are useful to indicate the relative quantity of antioxidant compounds solubilized by NaDES systems, and hence, of the extracted phenolics. Following this approach, the same evaluations were repeated after lignin precipitation, to estimate the residual amount of antioxidants.

Firstly, the radical scavenging activity was assessed for the as prepared biosolvents. While NaDESs showed complete absence of activity, LigDESs possess very low EC50 values, expressing intense antioxidant power (Table 2). Graphs and equations of the Probit regression of pure LigDES systems are reported in the SI (see Figures S8–S10).

**Figure 5:**
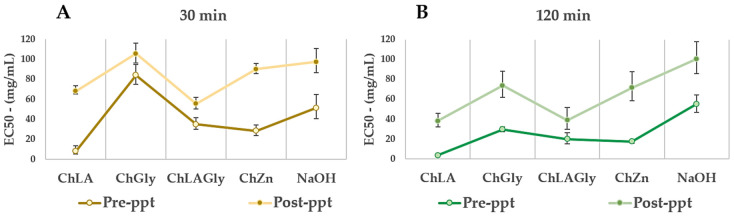
NaDES antioxidant power, expressed as EC50, pre- and post-lignin precipitation. (**A**): 30 min of treatment and (**B**): 120 min of treatment.

Considering these results, it was not possible to detect appreciable differences between LigDES alone and the extracts obtained after MW-assisted wheat straw pretreatment, thus excluding the latter from further evaluation. Conversely, in Figure 5A,B EC50 for the extracts obtained using NaDESs as solvents, both before and after lignin precipitation, are reported. As expected, longer extraction times (Figure 5B) resulted in lower EC50 values, probably due to the higher depolymerization degree leading to a higher content in antioxidant compounds.

It is worthy to note that all the extracts showed better antioxidant properties (that increase with increasing extraction times) than that obtained under conventional alkaline conditions, except for the one extracted using ChGly for 30 min. Indeed, ChGly was not only less effective in terms of extraction yields (Figure 3) but also considering the antioxidant properties of the extracts.

Studies on lignin model compounds have indicated that free phenolic hydroxyl groups (OHphen) and o-OCH_3_ substitution in the aromatic ring are essential for the antioxidant activity [49]. It can be hypothesized that ChLA, ChLAGly and ChZn allowed obtaining fractions with a higher content of these essential functional groups. High molecular weight, polydispersity and the presence of non-lignin impurities and heterogeneity are factors supposed to decrease radical scavenging activity. Indeed, the nonphenolic carbohydrate impurities, which remain strongly associated with lignin during its isolation and purification, can decrease the concentration of active OHphen groups and negatively influence their reactivity. Although the antioxidant properties of the extracts decrease after lignin precipitation, a certain radical scavenging activity is still observed, probably due to the presence of water-soluble antioxidant compounds. Low molecular weight phenols can also influence this behaviour [50].

### 2.5. Enzymatic Hydrolysis (EH)

**Figure 6:**
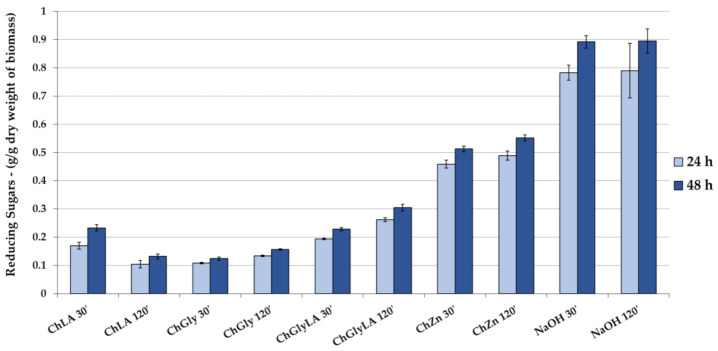
Enzymatic hydrolysis (EH) of solid fractions, resulting from microwave (MW)-assisted treatment of NaDESs. Yields are expressed as reducing sugars for 24 h and 48 h. Sample treated with NaOH is reported as a benchmark. Y-axis refers to dry delignified biomass.

**Figure 7:**
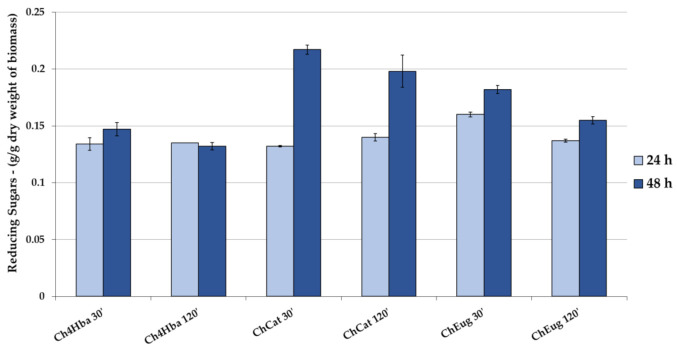
EH of solid fractions, resulting from LigDES MW-assisted treatment. Yields are expressed as reducing sugars for 24 h and 48 h. Y-axis refers to dry delignified biomass.

After the MW-assisted treatment of WS, the derived solid fraction was exploited for the enzymatic hydrolysis (EH). The results, expressed as reducing sugars’ content, are reported in Figure 6 and Figure 7, for NaDESs and LigDESs, respectively. The effect of the enzymatic reaction time on the hydrolysis efficiency was first evaluated. The pretreated wheat straws were mixed with a mixture of cellulase and beta-glucosidase enzymes for 24–48 h and the concentration of released reducing sugars was measured. The results showed that as the enzymatic reaction time increased, the concentration of the released reducing sugars slightly increased. This might be due to the inhibition of the enzyme activity by the accumulated hydrolysis products, which limit the effect of the reaction time on the yields. Among the conventional NaDESs, the best sugar yield was observed for the ChZn treated WS sample (after 120 min of MW irradiation) enabling a 0.55 of reducing sugar yields (g/g of dry delignified biomass) (Figure 6). This solvent was also very efficient in solubilizing lignin (Figure 3). However, the results on the biomass pretreated with ChLA were much worse, despite the high lignin extraction yields. This may be attributed to the effect of acidic NADES on the cellulase enzymes. Indeed, Kumar et al. demonstrated that, even after vigorous washing of the pretreated biomass with water for removal of the NADES reagent from the biomass residue, a still negligible amount of the NADES reagent was strongly bound to the biomass. Intriguingly, the cellulase activities were found to be increased significantly where the pH of the NADES reagent is close to neutrality, while it decreased in acidic NADES reagents, with a nearly complete loss in enzyme activity after 24-h incubation even in 5% (*v/v*) NADES reagent [42].

Enzymatic hydrolysis yields of 20% (around 0.2 g/g of dry delignified biomass) were described for the WS sample treated using LigDESs, with slightly better results recorded for ChCat (after 30 min of MW irradiation) (Figure 7). In this case, the increasing reaction time of EH strongly affected the yields in reducing sugars. These results require a more in-depth study to address the practical issues that arise especially in bioconversion processes using LigDES as an effective green solvent for lignin extraction.

### 2.6. Lignin-Derived Natural Deep Eutectic Solvents (LigDESs)

**Figure 8:**
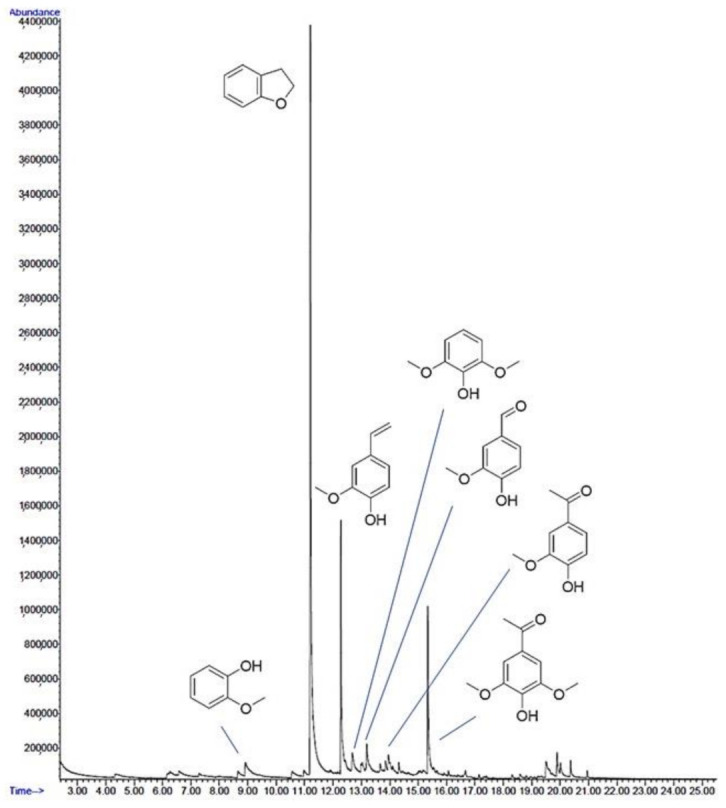
GC–MS chromatogram of liquid fraction after lignin precipitation.

According to the results reported in Section 2.3, the liquid fraction after lignin precipitation still contains residual antioxidant compounds. As a preliminary investigation, the liquid fraction remaining after the precipitation at acidic pH of soda lignin was analysed by GC–MS, in order to evaluate the composition. As documented in Figure 8, the main constituents of this fraction are phenolic monomers, therefore, they were recovered and recycled for the production of a new LigDES, aiming to the total biomass valorisation.

**Figure 9:**
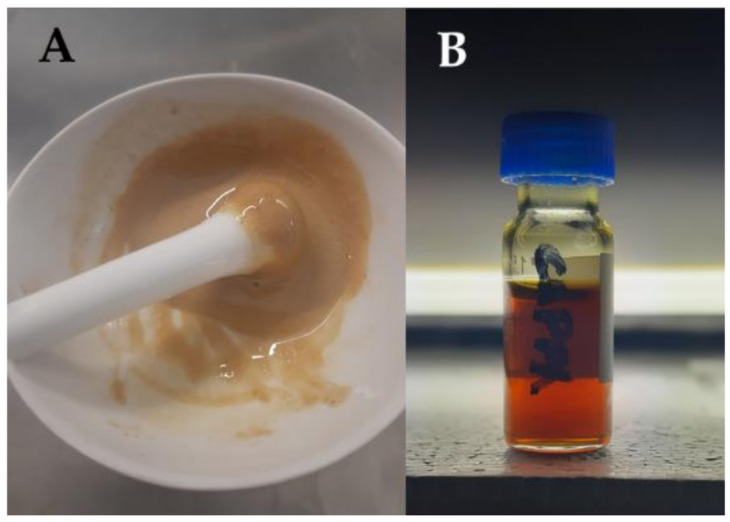
Image of the new DES obtained recycling the liquid fraction of wheat straw (WS) extract after lignin precipitation: (**A**) preparation and (**B**) obtained ChPPh (choline chloride:polyphenols mix).

In particular, this mixture was blended with ChCl (Figure 9A), until a stable, transparent brownish liquid was obtained: the ChPPh (choline chloride:polyphenols mix, see Section 3.2) (Figure 9B).

**Figure 10:**
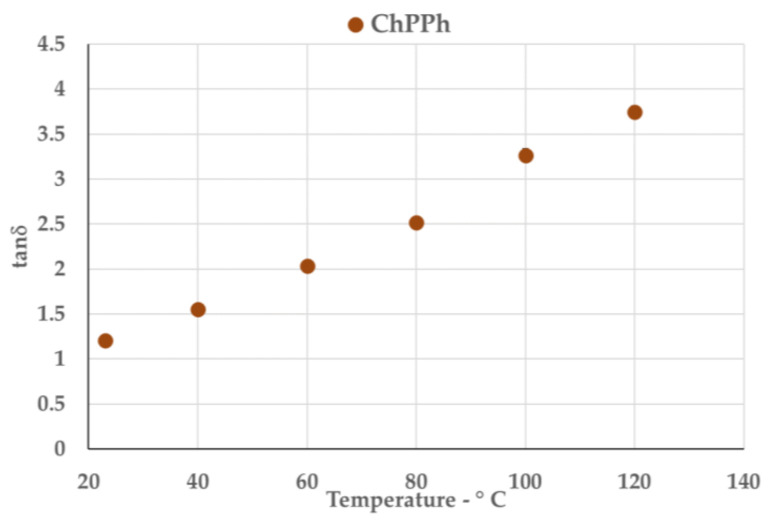
Variation of tan δ (@2.45 GHz) according to temperature for ChPPh.

This new solvent was then characterized for its dielectric properties; in particular, Figure 10 reports tan δ values in correlation with temperature increase, at 2.45 GHz, showing the high potential of this new LigDES in synergy with MW-assisted processes.

The variation of this parameter according to frequency sweep at 120 °C is reported in SI (see Figure S11).

The antioxidant power of pure ChPPh was evaluated (as reported for previous samples in Section 2.3), achieving a promising average EC50 of 7.3048 (6.101–8.805) mg/mL (relative graph and equation of Probit regression are reported in SI, see Figure S12).

## 3. Materials and Methods

### 3.1. Biomass

Wheat straw, WS, was provided in milled form (0.2 mm) by Environmental System GmbH (Bremerhaven, Germany). The matrix was stored in a dry place until use. WS consists of 21.8% (*w/w*) of total lignin and 8.5% (*w/w*) of inorganic materials (ashes). Further details are provided in the SI (see Table S1).

### 3.2. Chemicals and NaDES/LigDES Preparation

All chemicals were purchased from Sigma-Aldrich and used without further purification. NaDESs and LigDESs were synthetized via heating and stirring approach. ChCl was used as HBA for every investigated system. Conversely, different HBD molecules are explored, respecting the required molar ratio, as reported in Table 1 in Section 2.1. The mixtures were stirred for 1 h at 70 °C, until homogeneous liquids were obtained (see Figures S13 and S14). NaDESs and LigDESs were collected for WS delignification without further purification.

ChPPh was synthetized from the phenols mix recovered from NaOH extraction (120 min), after lignin precipitation (see Section 3.4.). The resulting liquid fraction was added of NaOH (2N) up to pH 7 and then freeze-dried. The sample was dissolved in MeOH (200 µL) and 1 µL was analysed by GC–MS with GC Agilent 6890 (Agilent Technologies, Santa Clara, CA, USA), fitted with an Agilent Network 5973 mass detector, using an HP-5MS 5% phenyl methyl siloxane column (30 m long capillary column, an i.d. of 0.25 mm and a film thickness of 0.25 μm). GC conditions were as follows; injection split 1:20, injector temperature 250 °C and detector temperature 280 °C. The gas carrier was helium (1.2 mL/min), and the temperature program proceeded from 70 (2 min) to 300 °C at a rate of 5 °C/min.

The dry material was blended in a mortar with increasing quantity of ChCl, following fixed g/g ratios. The final ratio of 3:1 (*w/w*, ChCl:polyphenols mix) led to a turbid solution, clarified by centrifugation (26,000 rpm, 1 min, Allegra 64R Centrifuge, Beckman Coulter, Brea, CA, USA). The transparent, amber-coloured liquid was stored for analysis.

### 3.3. Dielectric Properties of Deep Eutectic Solvents (NaDES and LigDES)

Dielectric properties of the NaDESs were measured by the open ended coaxial probe technique in the frequency range 0.3–3 GHz, from room temperature up to 120 °C (corresponding to the employed temperature during MW assisted experiments), by means of an Agilent 8753C vector network analyser connected to an Agilent 85070E dielectric kit probe. During these measurements, the temperature was monitored by an optical fibre (Neoptix Reflex four channels, temperature range −50, +250 °C, Qualitrol company, Québec, QC, Canada) directly inserted into the sample. The measurements were performed in triplicate during the heating of the specimen at ca. every 20 °C step.

### 3.4. MW-Assisted Wheat Straw (WS) Delignification

MW-assisted delignification was performed by means of a multimode MW reactor (SynthWAVE Milestone Srl, Bergamo, Italy). The system runs with a maximum power of 1500 W, at a working frequency of 2.45 GHz. Every biobased solvent (NaDES and LigDES) was tested for WS delignification in a solid/liquid ratio of 1:15 (*w/w*), treating 1 g of biomass at a time. The process was performed at 120 °C, for 30 and 120 min, under 500 rpm of stirring. For the sake of comparison, a NaOH system (10% on dry WS) was tested in the same conditions. Before every run, the reactor was purged three times with N_2_ to avoid oxidative degradations. The same gas was applied to pressurize the system up to 2 bars, avoiding water traces to boil.

After the MW irradiation, the mixture was centrifuged three times for 30 min at 4000 rpm (Rotofix 32, Hettich GmbH, Tuttlingen, Germany). The liquid fraction was recovered and stored for analysis. Residual biomass was washed thoroughly with deionized water and vacuum-filtered, until the eutectic solvent was completely removed. Further washings with ethanol were necessary to remove from WS the residues of eugenol and 4-hydroxybenzyl alcohol, only partially soluble in water. The recovered solid fraction was dried under vacuum and stored for EH.

A different separation strategy was adopted for ChZn systems, due to solution texture. In detail, 10 mL of GVL were added for every centrifugation step (for a total 30 mL addition). The resulting liquid fraction was stored for analysis, taking into account the derived dilution. Finally, solid fraction underwent the abovementioned work-up.

### 3.5. Lignin Precipitation and NaDES Recovery

The lignin dissolved by the eutectic systems during the process was recovered via precipitation. In detail, the framework of ChLA, ChGly, ChLAGly, ChEug, ChCat and Ch4Hba was suppressed by water addition. 75% (*w/w*) of distilled water was added to approximately 1 g of DES liquid fraction. Conversely, ChZn/GVL fractions were separated from lignin by antisolvent procedure, keeping a 9:1 ratio of water addition, according to a previous work [12]. Thirdly, lignin was precipitated from alkaline solutions by acidification. In detail concentrated H_2_SO_4_ was added to reach pH 2, according a previously presented protocol [17].

The precipitation process was accelerated by means of ultracentrifugation (26,000 rpm, 5 min, Allegra 64R Centrifuge, Beckman Coulter, CA, USA), performed at RT. The resulting solid underwent a washing/centrifuging cycle for three times with distilled water and, in the case of ChEug and Ch4Hba, with ethanol. Finally, the recovered precipitate was dried under vacuum and weighted.

The liquid fraction resulting from the separation protocol (see Figure S15) was freeze-dried to remove excess water and tested for the residual antioxidant power. Water and pH adjustments were performed where required.

### 3.6. Antioxidant Activity of Recovered Liquid Fraction—DPPH Essay

The radical scavenging activity of the liquid fractions was evaluated using the free radical DPPH, according to the method already exploited in a previous work [37]. The bleaching rate of the DPPH was monitored to calculate the EC50 (half maximal effective concentration or amount of compound/extract necessary to decrease the initial concentration of DPPH to 50% at equilibrium). Various concentrations of liquid samples were analysed at 515 nm (Cary 60 UV-VIS spectrophotometer, Agilent Technologies, Santa Clara, CA, USA). Collected data were processed by Bobo Least Squares software (ver. 0.9.1.) to establish an accurate Probit regression (R ≥ 0.96). All the samples were analysed in triplicate, and the DPPH radical scavenging activity was expressed as mg of liquid fraction/mL solution and the relative confidence interval.

### 3.7. Enzymatic Hydrolysis (EH)

Hydrolysis was performed in 100 mL flasks with 20 mL of 50 mM acetate buffer pH 5.0 and 1% of substrate concentration. A mixture of *Trichoderma reesei* 101 and *Aspergillus awamori* enzymes was used for enzymatic hydrolysis, being a source of cellulases and the beta-glucosidase respectively. The dose of enzymes was 15 mg protein/g substrate (calculated on the basis of endocellulase activity, 9330.75 nkat/g substrate) and beta-glucosidase was 200 nkat/g substrate. To inhibit microbiological growth, 0.05% NaN_3_ was added. The flasks were incubated for 48 h at 45 °C and stirred at 150 rpm.

Soluble sugars in the samples were determined by estimating the amount of reduced sugars in the soluble fraction using the 2-hydroxy-3,5-dinitrobenzoic acid (DNS) method against glucose standards [51].

## 4. Conclusions

Aiming to solve the bottlenecks of current biomass pretreatment step, greener solvents and MW technology were coupled herein to achieve a sustainable lignocellulosic biomass delignification. The study shows that DESs are effective at selectively dissolving lignin in biomass fractionation. MW heating enhances this process by faster heating. In addition, lignin-derived DESs were characterized and tested for MW-assisted lignin extraction, showing promising performance, meanwhile the solid cellulose-rich fraction was proved suitable for enzymatic saccharification. Further detailed investigation on these research areas will provide the versatile uses of NaDESs for significantly lower cost and ecofriendly processes.

Moreover, a new lignin derived NaDES, the ChPPh, was prepared starting from the recovered liquid fraction after lignin precipitation from wheat straw extracts. It shows excellent dielectric properties and could be a promising solvent for a wide range of MW-assisted applications. This study enabled the full valorisation of all the biomass components, according to a zero-waste approach, paving the way to circular loop of biorefinery processes.

## Figures and Tables

**Table 1:** Natural deep eutectic solvent (NaDES) and lignin derived NaDES (LigDES). Abbreviations, hydrogen bonding donor (HBD) structures and molar ratios. Water addition is expressed as *w/w* percentage.

Natural Deep Eutectic Solvents (NaDESs)	HBD	HBA/HBD Molar Ratio
ChLA	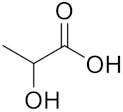	1:10
ChGly	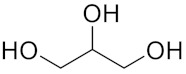	1:2
ChGlyLA	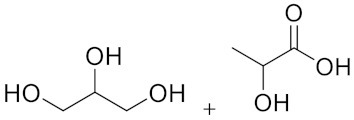	1:1:1
ChZn	ZnCl_2_	1:2 (+10% H_2_O)
**Lignin-Derived Deep Eutectic Solvents (LigDESs)**	**HBD**	**HBA/HBD Molar Ratio**
Ch4Hba	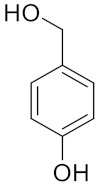	1:1
ChCat	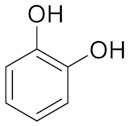	1:1
ChEug	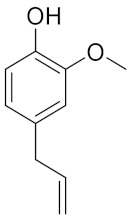	1:1 (+10% H_2_O)

**Table 2:** Antioxidant activity of as prepared LigDES systems and half maximal effective concentration or amount of compound/extract necessary to decrease the initial concentration of DPPH to 50% at equilibrium (EC50) determination by means of DPPH assay.

Lignin-Derived Deep Eutectic Solvents (LigDESs)	EC50 (mg/mL)	
Ch4Hba	1.9758	1.1103–3.7188
ChCat	0.1065	0.0741–0.1616
ChEug	0.3148	0.2094–0.4774

## Data Availability

The data presented in this study are available in supplementary materials.

## Supplementary Materials

The following are available online, Figure S1: ChLA dielectric properties; Figure S2: ChGly dielectric properties; Figure S3: ChGlyLA dielectric properties; Figure S4: ChZn dielectric properties; Figure S5: Ch4Hba dielectric properties; Figure S6: ChCat dielectric properties; Figure S7: ChEug dielectric properties; Figure S8: Ch4Hba “as synthetized” DPPH essay; Figure S9: ChCat “as synthetized” DPPH essay; Figure S10: ChEug “as synthetized” DPPH essay; Figure S11: ChPPh dielectric properties; Figure S12: ChPPh “as synthetized” DPPH essay; Figure S13: Choline based NaDES; Figure S14: Choline based LigDES; Figure S15: Choline based NaDES after lignin precipitation and water excess elimination, Table S1: WS biomass characterization.

## References

[1] Clarke C.J., Tu W.-C., Levers O., Bröhl A., Hallett J.P. (2018). Green and Sustainable Solvents in Chemical Processes. Chem. Rev..

[2] Costa Lopes A.M., Lins R.M.G., Rebelo R.A., Łukasik R.M. (2018). Biorefinery approach for lignocellulosic biomass valorisation with an acidic ionic liquid. Green Chem..

[3] Calcio Gaudino E., Cravotto G., Manzoli M., Tabasso S. (2019). From waste biomass to chemicals and energy via microwave-assisted processes. Green Chem..

[4] Rosales-Calderon O., Arantes V. (2019). A review on commercial-scale high-value products that can be produced alongside cellulosic ethanol. Biotechnol. Biofuels.

[5] Rinaldi R., Jastrzebski R., Clough M.T., Ralph J., Kennema M., Bruijnincx P.C.A., Weckhuysen B.M. (2016). Paving the Way for Lignin Valorisation: Recent Advances in Bioengineering, Biorefining and Catalysis. Angew. Chem. Int. Ed..

[6] Kumar A.K., Sharma S. (2017). Recent updates on different methods of pre-treatment of lignocellulosic feedstocks: A review. Bioresour. Bioprocess..

[7] Bello S., Rios C., Feijoo G., Moreira M.T. (2018). Comparative evaluation of lignocellulosic biorefinery scenarios under a life-cycle assessment approach. Biofuels Bioprod. Biorefin..

[8] Yang B., Wyman C.E. (2007). Pretreatment: The key to unlocking low-cost cellulosic ethanol. Biofuels Bioprod. Biorefin..

[9] Cravotto G., Carnaroglio D. (2017). Microwave Chemistry.

[10] Clark J.H., Farmer T.J., Herrero-Davila L., Sherwood J. (2016). Circular Economy Design Considerations for Research and Process Development in the Chemical Sciences. Green Chem..

[11] Shuai L., Questell-Santiago Y.M., Luterbacher J.S. (2016). A mild biomass pretreatment using γ-valerolactone for concentrated sugar production. Green Chem..

[12] Tabasso S., Grillo G., Carnaroglio D., Calcio Gaudino E., Cravotto G. (2016). Microwave-Assisted γ-Valerolactone Production for Biomass Lignin Extraction: A Cascade Protocol. Molecules.

[13] Li S.-X., Li M.F., Bian J., Sun S.-N., Peng F., Xue Z.M. (2017). Biphasic 2-methyltetrahydrofuran/oxalic acid/water pretreatment to enhance cellulose enzymatic hydrolysis and lignin valorization. Bioresour. Technol..

[14] Cai C.M., Zhang T., Kumar R., Wyman C.E. (2013). THF co-solvent enhances hydrocarbon fuel precursor yields from lignocellulosic biomass. Green Chem..

[15] Gschwend F.J.V., Malaret F., Shinde S., Brandt-Talbot A., Hallett J.P. (2018). Rapid pretreatment of Miscanthus using the low-cost ionic liquid triethylammonium hydrogen sulfate at elevated temperatures. Green Chem..

[16] Kim K.H., Dutta T., Sun J., Simmons B., Singh S. (2018). Biomass pretreatment using deep eutectic solvents from lignin-derived phenols. Green Chem..

[17] Calcio Gaudino E., Tabasso S., Grillo G., Cravotto G., Dreyer T., Schories G., Altenberg S., Lauberte L., Telysheva G. (2018). Wheat straw lignin extraction with bio-based solvents using enabling technologies Comptes Rendus Chem. Comptes Rendus Chem..

[18] Brandt A., Gräsvik J., Hallett J.P., Welton T. (2013). Deconstruction of lignocellulosic biomass with ionic liquids. Green Chem..

[19] Gräsvik J., Winestrand S., Normark M., Jönsson L.J., Mikkola J.P. (2014). Evaluation of four ionic liquids for pretreatment of lignocellulosic biomass. BMC Biotechnol..

[20] Hajipour A.R., Rafiee F. (2015). Recent Progress in Ionic Liquids and their Applications in Organic Synthesis. Org. Prep. Proced. Int..

[21] Martins M.A.P., Frizzo C.P., Moreira D.N., Zanatta N., Bonacorso H.G. (2008). Ionic Liquids in Heterocyclic Synthesis. Chem. Rev..

[22] Tadesse H., Luque R. (2011). Advances on biomass pretreatment using ionic liquids: An overview. Energy Environ. Sci..

[23] Abbott A.P., Boothby D., Capper G., Davies D.L., Rasheed R.K. (2004). Deep eutectic solvents formed between choline chloride (Ch) and carboxylic acids: Versatile alternatives to ionic liquids. J. Am. Chem. Soc..

[24] Smith E.L., Abbott A.P., Ryder K.S. (2014). Deep Eutectic Solvents (DESs) and their applications. Chem. Rev..

[25] Mbous Y.P., Hayyan M., Hayyan A., Wong W.F., Hashim M.A., Looi C.Y. (2017). Applications of deep eutectic solvents in biotechnology and bioengineering—promises and challenges. Biotechnol. Adv..

[26] Xu G.-C., Ding J.-C., Han R.-Z., Dong J.-J., Ni Y. (2016). Enhancing cellulose accessibility of corn stover by deep eutectic solvent pretreatment for butanol fermentation. Bioresour. Technol..

[27] Gorke J.T., Srienc F., Kazlauskas R.J. (2010). Deep eutectic solvents for *Candida antarctica* lipase B-catalyzed reactions. In: Ionic Liquid Applications: Pharmaceuticals, Therapeutics, and Biotechnology. ACS Symp. Ser..

[28] Oliveira V.K.D., Gregory C., Francois J. (2015). Contribution of deep eutectic solvents for biomass processing: Opportunities, challenges, and limitations. ChemCatChem.

[29] Chen Y., Mu T. (2019). Application of deep eutectic solvents in biomass pretreatment and conversion. Green Energy Environ..

[30] Van Osch D.J.G.P., Kollau L.J.B.M., van den Bruinhorst A., Asikainen S., Rocha M.A.A., Kroon M.C. (2017). Ionic liquids and deep eutectic solvents for lignocellulosic biomass fractionation. PCCP.

[31] Suopajärvi T., Ricci P., Karvonen V., Ottolina G., Liimatainen H. (2020). Acidic and alkaline deep eutectic solvents in delignification and nanofibrillation of corn stalk, wheat straw, and rapeseed stem residues. Ind. Crops Prod..

[32] Choi Y.H., van Spronsen J., Dai Y., Verberne M., Hollmann F., Arends I.W.C.E., Witkamp G.-J., Verpoorte R. (2011). Are natural deep eutectic solvents the missing link in understanding cellular metabolism and physiology?. Plant Physiol..

[33] Dai Y., Witkamp G.J., Verpoorte R., Choi Y.H. (2013). Natural deep eutectic solvents as new extraction media for phenolic metabolites in safflower. Anal. Chem..

[34] Durand E., Lecomte J., Barea B., Dubreucq E., Lortie R., Villeneuve P. (2013). Evaluation of deep eutectic solvent-water binary mixtures for lipase-catalyzed lipophilization of phenolic acids. Green Chem..

[35] Zhao H., Baker G.A., Holmes S. (2011). New eutectic ionic liquids for lipase activation and enzymatic preparation of biodiesel. Org. Biomol. Chem..

[36] Grillo G., Gunjević V., Radošević K., Radojčić Redovniković I., Cravotto G. (2020). Deep Eutectic Solvents and Nonconventional Technologies for Blueberry-Peel Extraction: Kinetics, Anthocyanin Stability, and Antiproliferative Activity. Antioxidants.

[37] Samorì C., Mazzei L., Ciurli S., Cravotto G., Grillo G., Guidi E., Pasteris A., Tabasso S., Galletti P. (2019). Urease inhibitory potential and soil ecotoxicity of novel “polyphenols-deep eutectic solvents” formulations. ACS Sustain. Chem. Eng..

[38] Ivanović M., Islamčević Razboršek M., Kolar M. (2020). Innovative Extraction Techniques Using Deep Eutectic Solvents and Analytical Methods for the Isolation and Characterization of Natural Bioactive Compounds from Plant Material. Plants.

[39] Dai Y., Verpoorte R., Choi Y.H. (2014). Natural deep eutectic solvents providing enhanced stability of natural colorants from safflower (*Carthamus tinctorius*). Food Chem..

[40] Tan Y.T., Chua A.S.M., Ngoh G.C. (2020). Deep eutectic solvent for lignocellulosic biomass fractionation and the subsequent conversion to bio-based products—A review. Bioresour. Technol..

[41] Wang Z.-K., Li H., Lin X.-C. (2020). Novel recyclable deep eutectic solvent boost biomass pretreatment for enzymatic hydrolysis. Bioresour. Technol..

[42] Kumar A.K., Sharma S., Dixit G., Shah E., Patel A. (2020). Techno-economic evaluation of a natural deep eutectic solvent-based biorefinery: Exploring different design Scenarios. Biofuels Bioprod. Bioref..

[43] Kumar A.K., Parikh B.S., Pravakar M. (2016). Natural deep eutectic solvent mediated pretreatment of rice straw: Bioanalytical characterization of lignin extract and enzymatic hydrolysis of pretreated biomass residue. Environ. Sci. Pollut. Res. Int..

[44] Dai Y., van Spronsen J., Witkamp G.-J., Verpoorte R., Choi Y.H. (2013). Natural deep eutectic solvents as new potential media for green technology. Anal Chim. Acta.

[45] Dai Y., Witkamp G.J., Verpoorte R., Choi Y.H. (2015). Tailoring properties of natural deep eutectic solvents with water to facilitate their applications. Food Chem..

[46] Muley P.D., Mobley J.K., Tong X., Novak B., Stevens J., Moldovan D., Shi J., Boldor D. (2019). Rapid microwave-assisted biomass delignification and lignin depolymerization in deep eutectic solvents. Energy Convers. Manag..

[47] Abbott A.P., Bell T.J., Handa S., Stoddart B. (2006). Cationic functionalisation of cellulose using a choline based ionic liquid analogue. Green Chem..

[48] Hou X.-D., Ao-Lin L., Lin K.-P., Wang Y.-Y., Kuang Z.-Y., Cao S.-L. (2018). Insight into the structure-function relationships of deep eutectic solvents during rice straw pretreatment. Bioresour. Technol..

[49] Dizhbite T., Telysheva G., Jurkjane V., Viesturs U. (2004). Characterization of the radical scavenging activity of lignins –natural antioxidants. Bioresour. Technol..

[50] Ponomarenko J., Lauberts M., Dizhbite T., Lauberte L., Jurkjane V., Telysheva G. (2015). Antioxidant activity of various lignins and lignin-related phenylpropanoid units with high and low molecular weight. Holzforschung.

[51] Bernfeld P. (1955). Amylase α and β. Methods Enzymol..

