# Supplementary material for: Green Deep Eutectic Solvents for Microwave-Assisted Biomass Delignification and Valorisation
Source: Molecules. 2021 Feb 4;26(4):798. doi: 10.3390/molecules26040798 (PMC7913847; doi:10.3390/molecules26040798)
Supplement: Supplementary file 1 [file molecules-26-00798-s001.pdf]

SUPPORTING INFO

Dielectric Properties of Deep Eutectic Solvents (NaDES and LigDES)

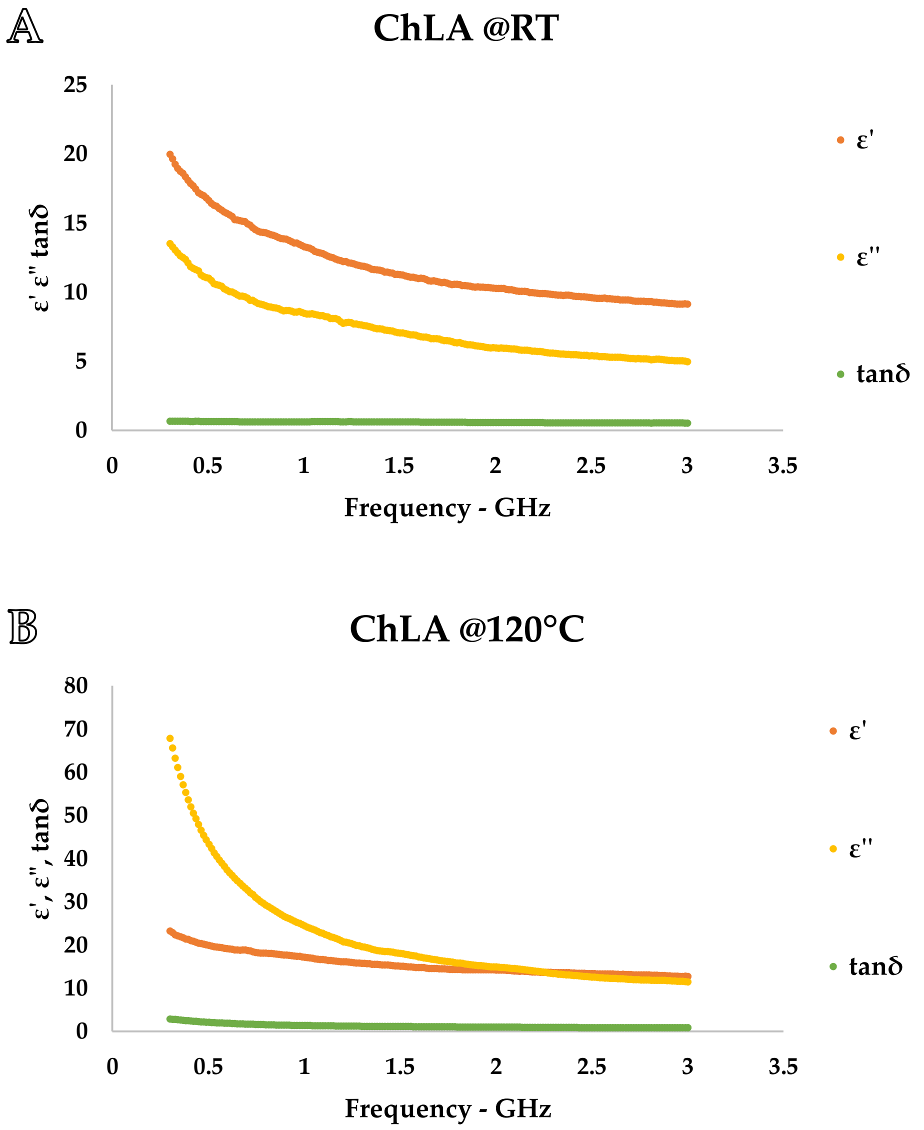

Figure S1: ChLA dielectric properties. Irradiation frequency range: 0.3-3 GHz. Irradiation temperature: A. RT; B. 120 °C. Measurements were performed according to Paragraph 3.3 in the Experimental Section.

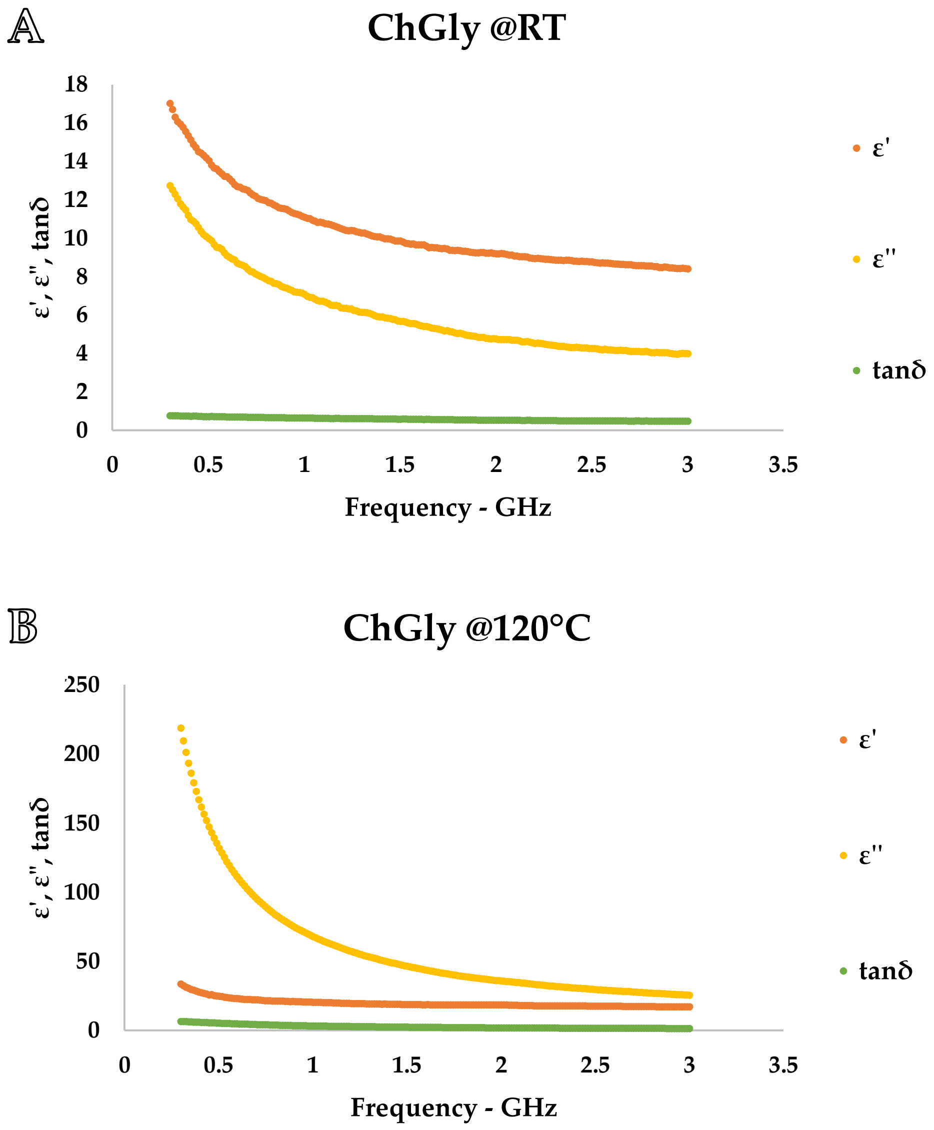

Figure S2: ChGly dielectric properties. Irradiation frequency range: 0.3-3 GHz. Irradiation temperature: A. RT; B. 120 °C. Measurements were performed according to Paragraph 3.3 in the Experimental Section.

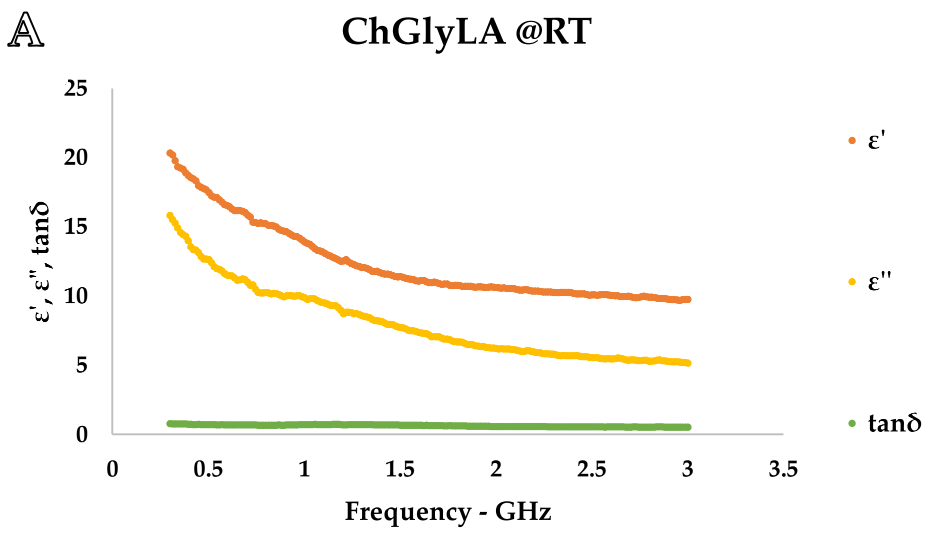

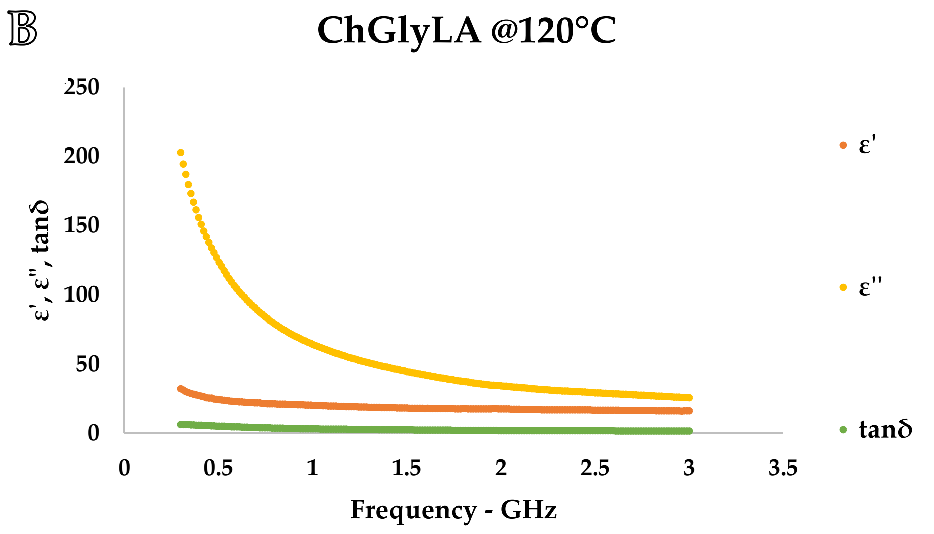

Figure S3: ChGlyLA dielectric properties. Irradiation frequency range: 0.3-3 GHz. Irradiation temperature: A. RT; B. 120 °C. Measurements were performed according to Paragraph 3.3 in the Experimental Section.

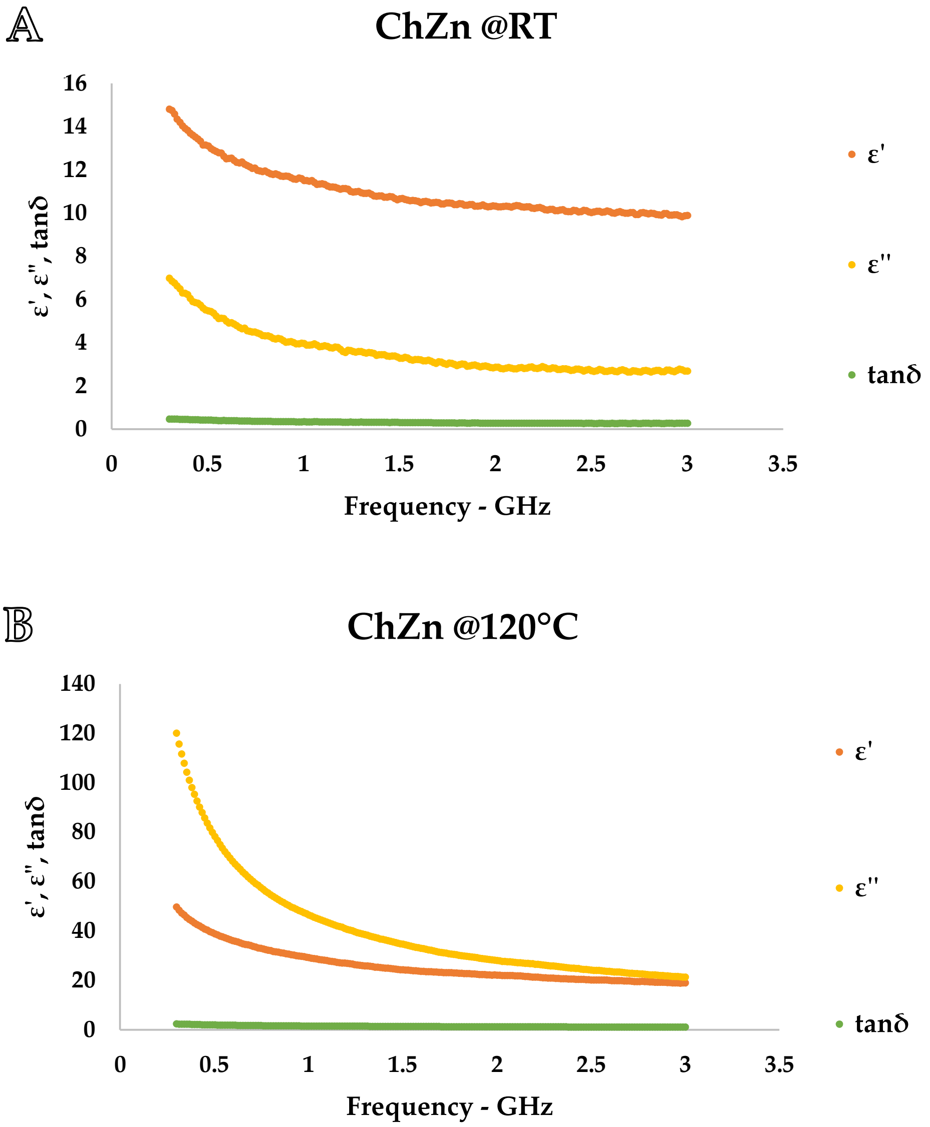

Figure S4: ChZn dielectric properties. Irradiation frequency range: 0.3-3 GHz. Irradiation temperature: A. RT; B. 120 °C. Measurements were performed according to Paragraph 3.3 in the Experimental Section.

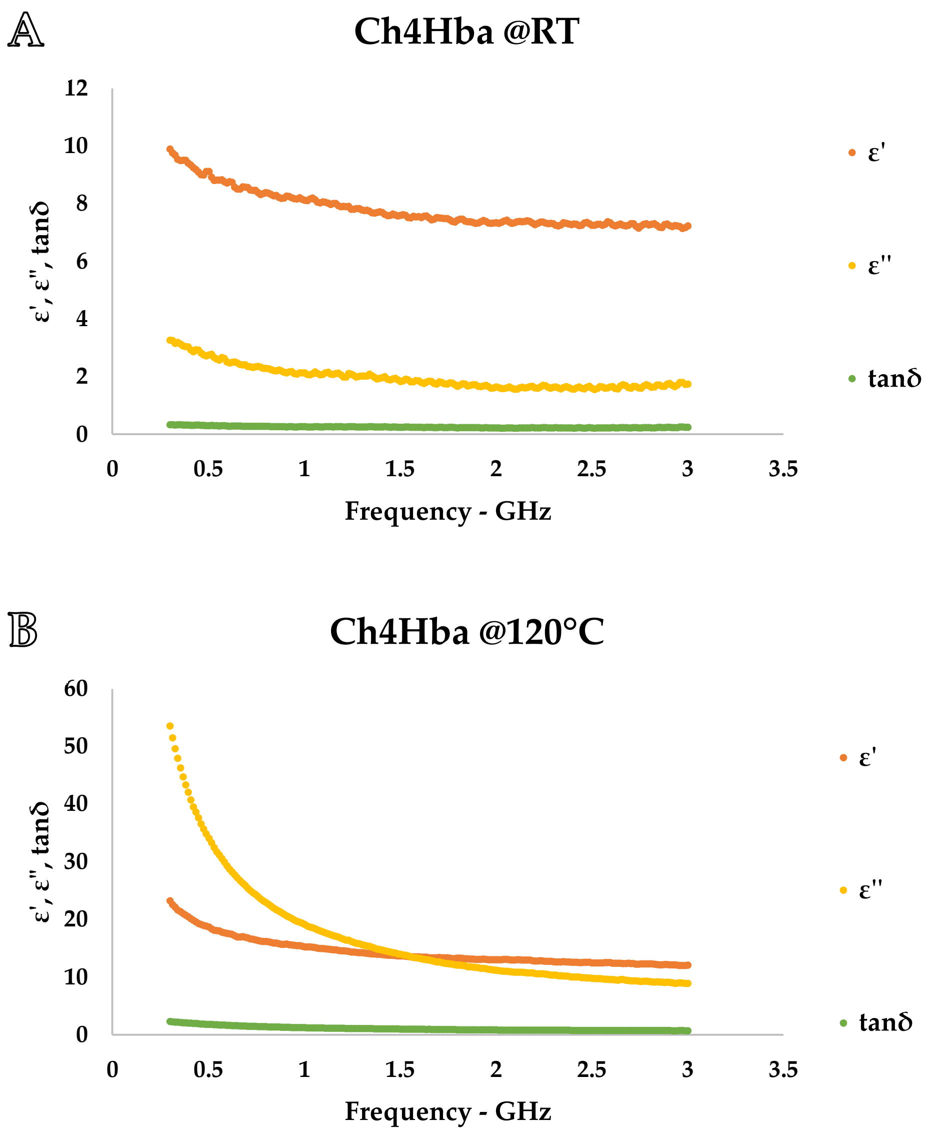

Figure S5: Ch4Hba dielectric properties. Irradiation frequency range: 0.3-3 GHz. Irradiation temperature: A. RT; B. 120 °C. Measurements were performed according to Paragraph 3.3 in the Experimental Section.

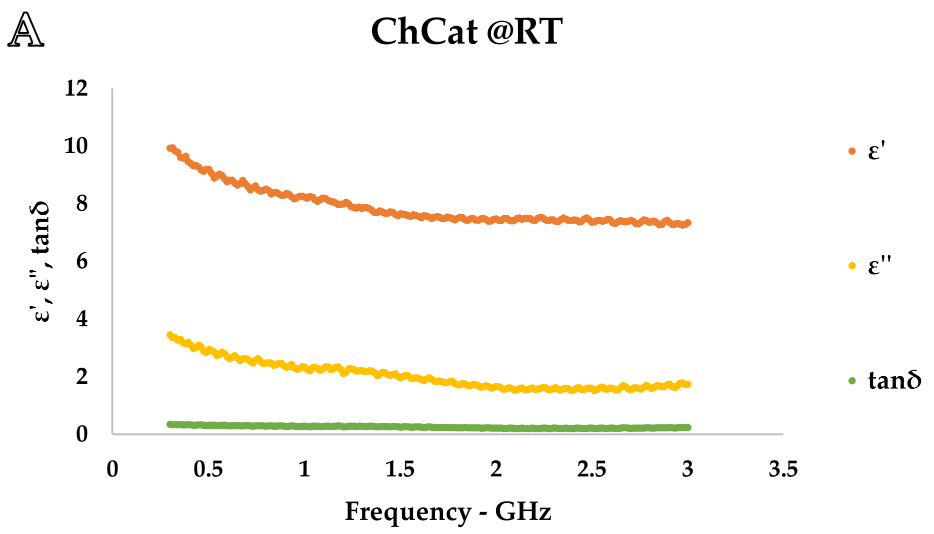

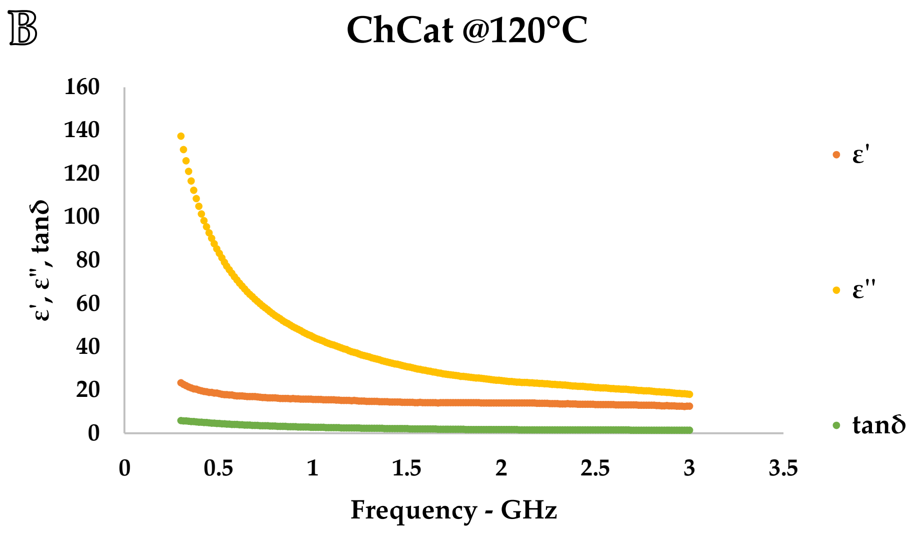

Figure S6: ChCat dielectric properties. Irradiation frequency range: 0.3-3 GHz. Irradiation temperature: A. RT; B. 120 °C. Measurements were performed according to Paragraph 3.3 in the Experimental Section.

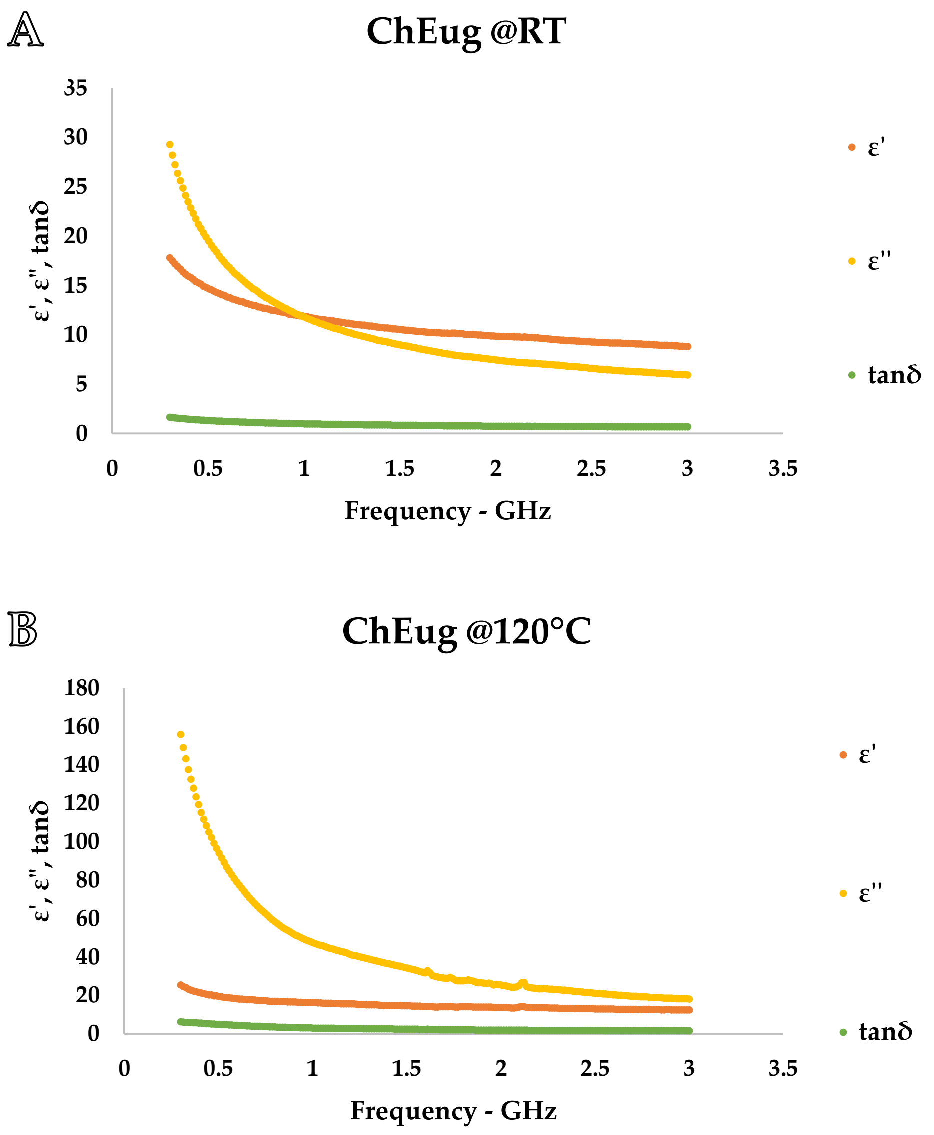

Figure S7: ChEug dielectric properties. Irradiation frequency range: 0.3-3 GHz. Irradiation temperature: A. RT; B. 120 °C. Measurements were performed according to Paragraph 3.3 in the Experimental Section.

Wheat straw Characterization

Table S1: WS biomass characterization, NREL method. [1]

		Carbohydrates (%, DM)	Lignin (%, DM)		
Ash (%, DM)	Extractives (%, DM)	TOT	Acid insol.	Acid sol.	TOT
8.5	0.69	73.2	20.6	1.2	21.8

Antioxidant Activity of LigDES

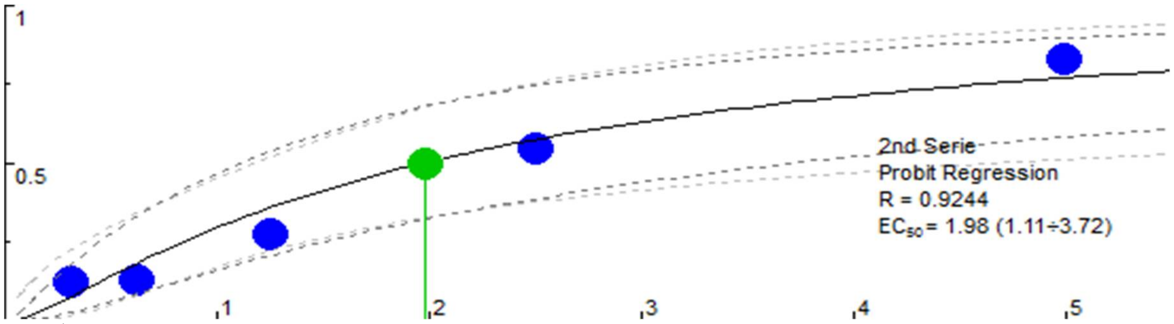

Probit Regression

Y = CumNorm(0.7962011 * Log(X) -0.5421771)

R = 0.92439

EC50 = 1.9758 (1.1103÷3.7188)

Figure S8: Ch4Hba "as synthetized" DPPH essay. Probit regression, relative equation and EC50 value. Measurements were performed according to Paragraph 3.6 in the Experimental Section.

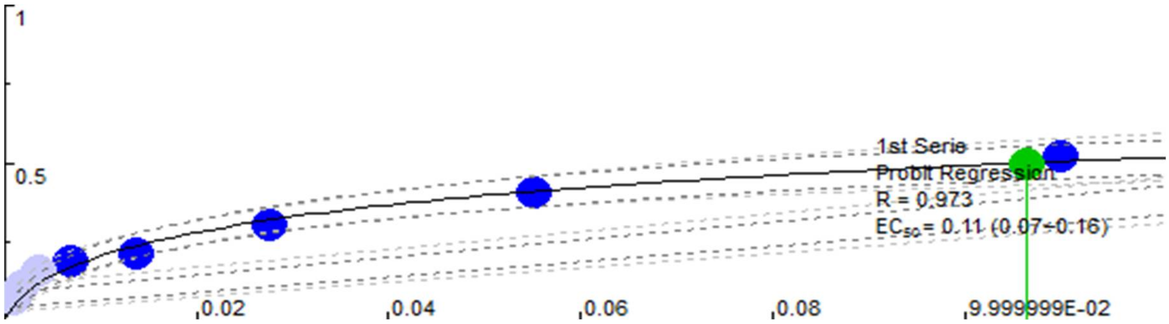

Probit Regression

Y = CumNorm(0.350742 * Log(X) + 0.785555)

R = 0.972972

EC50 = 0.1065 (0.0741÷0.1616)

Figure S9: ChCat "as synthetized" DPPH essay. Probit regression, relative equation and EC50 value. Measurements were performed according to Paragraph 3.6 in the Experimental Section.

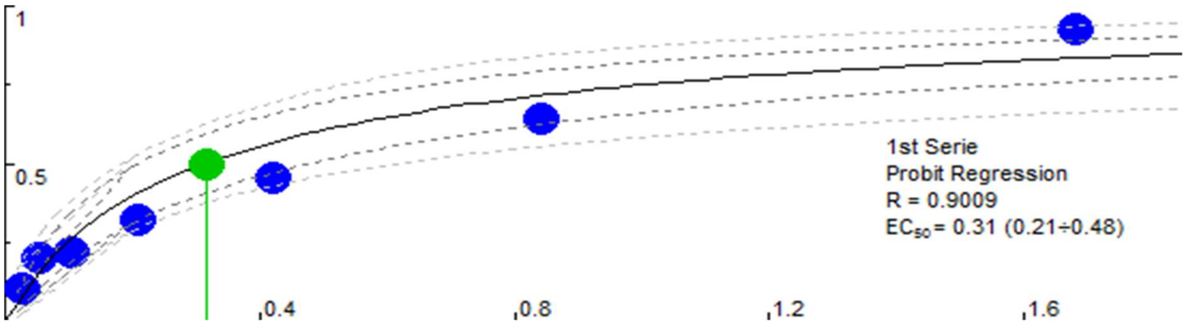

Probit Regression

Y = CumNorm(0.58189 * Log(X) + 0.67258)

R = 0.900917

EC50 = 0.3148 (0.2094÷0.4774)

Figure S10: ChEug "as synthetized" DPPH essay. Probit regression, relative equation and EC50 value. Measurements were performed according to Paragraph 3.6 in the Experimental Section.

Dielectric Properties of ChPPh LigDES

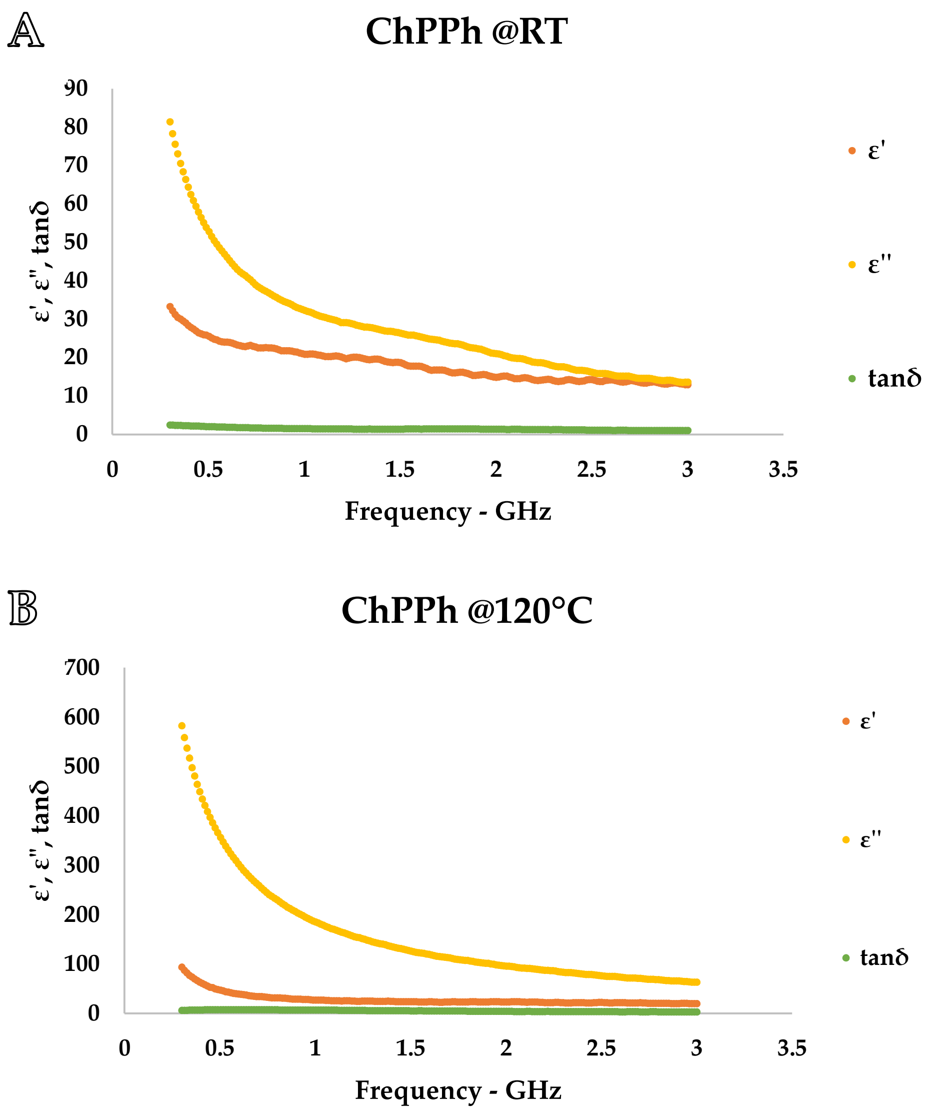

Figure S11: ChPPh dielectric properties. Irradiation frequency range: 0.3-3 GHz. Irradiation temperature: A. RT; B. 120 °C. Measurements were performed according to Paragraph 3.3 in the Experimental Section.

Antioxidant Activity of ChPPh LigDES

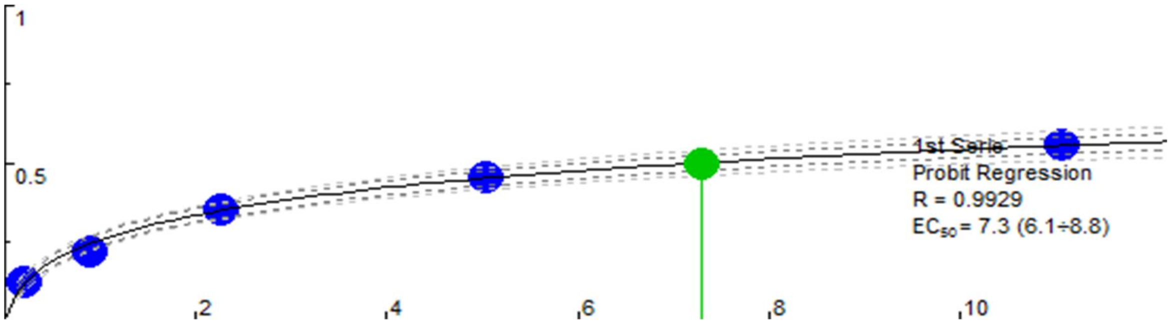

Probit Regression

Y = CumNorm(0.332142 * Log(X) -0.660476)

R = 0.992903

EC50 = 7.3048 (6.101÷8.805)

Figure S12: ChPPh "as synthetized" DPPH essay. Probit regression, relative equation and EC50 value. Measurements were performed according to Paragraph 3.6 in the Experimental Section.

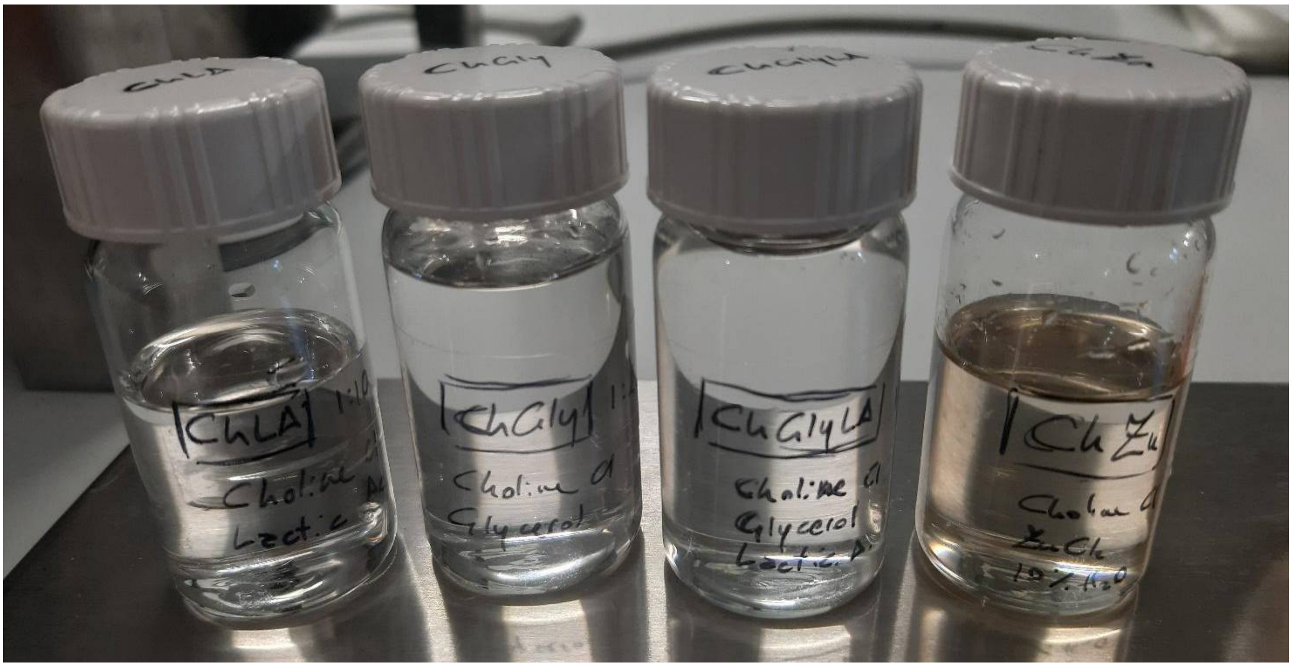

Figure S13: Choline based NaDES. HBD from left to right: lactic acid (LA), glycerol (Gly), lactic acid + glycerol (GlyLA), $ZnCl_2$ (Zn).

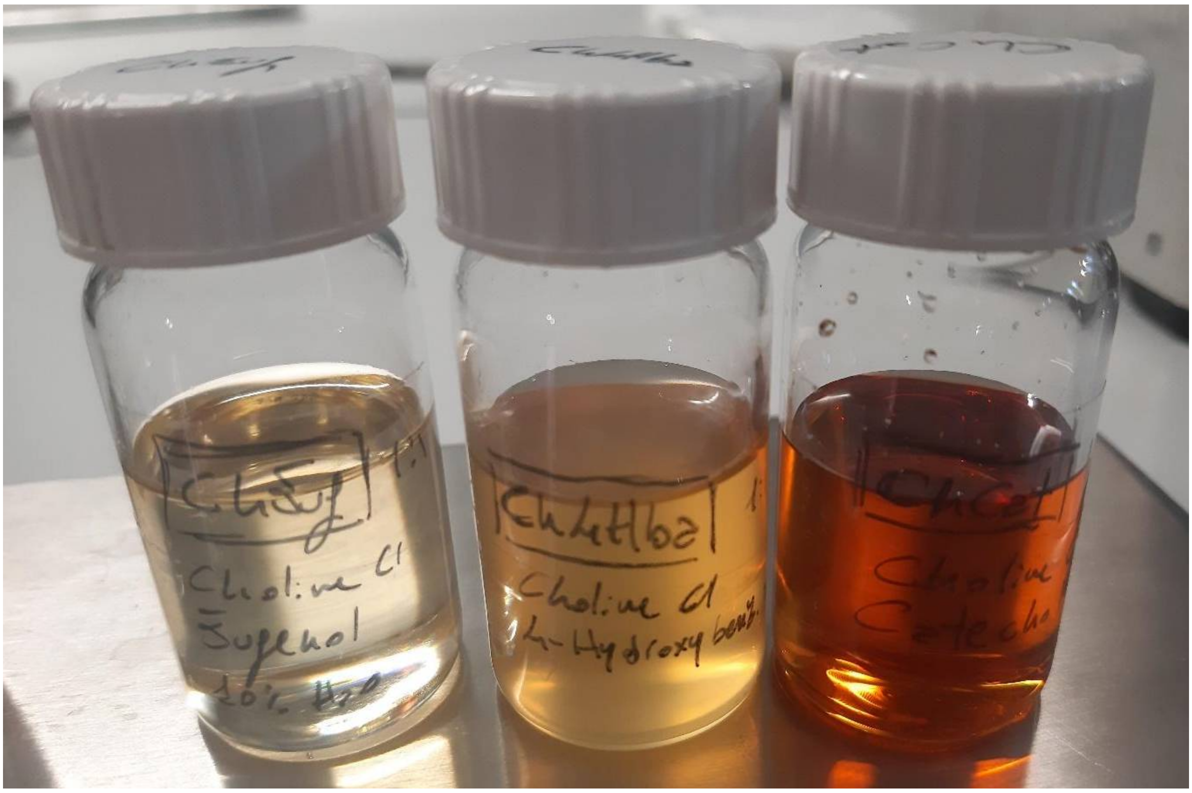

Figure S14: Choline based LigDES. HBD from left to right: eugenol (Eug), 4-hydroxybenzyl alcohol (4Hba), catechol (Cat).

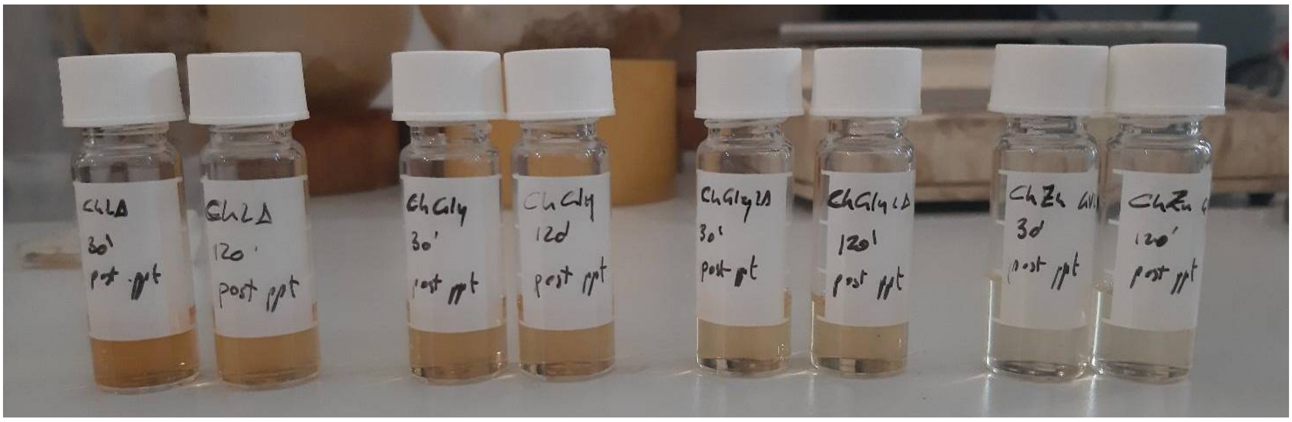

Figure S15: Choline based NaDES after lignin precipitation and water excess elimination. From left to right: ChLA 30 min and 120 min, ChGly 30 min and 120 min, ChGlyLA 30 min and 120 min, ChZn 30 min and 120 min.

References

1. Genevini, P.; Adani, F.; Villa, C.; Rice hull degradation by co-composting with dairy cattle slurry. *Soil Sci. Plant. Nutr.* **1997**, *43*, 135–147. https://doi.org/10.1080/00380768.1997.10414722.
